# Do the amplitude ratios of sensory nerve action potentials in the lower extremities have any diagnostic utility in distal diabetic polyneuropathy?

**DOI:** 10.55730/1300-0144.6014

**Published:** 2025-04-12

**Authors:** Işıl YAZICI GENÇDAL, Nermin Görkem ŞİRİN, İrem İLGEZDİ, Ümmü MUTLU, Elif KOCASOY-ORHAN, Mehmet Barış BASLO, Nevin DİNÇÇAĞ, Ali Emre ÖGE

**Affiliations:** 1 Division of Clinical Neurophysiology, Department of Neurology, Faculty of Medicine, İstanbul University, İstanbul, Turkiye; 2 Division of Endocrinology, Department of Internal Medicine, Faculty of Medicine, İstanbul University, İstanbul, Turkiye

**Keywords:** Medial femoral cutaneous sensory nerve action potential, dorsal sural sensory nerve action potential, sural/radial amplitude ratio, diabetic polyneuropathy, length-dependent axonal polyneuropathy

## Abstract

**Background/aim:**

To investigate the diagnostic sensitivity of sural sensory nerve action potential (SNAP) to medial femoral cutaneous nerve and dorsal sural to sural SNAP amplitude ratios in patients with diabetic polyneuropathy.

**Materials and methods:**

Sural/radial (SRAR), sural/medial femoral cutaneous (SMFAR), and dorsal sural/sural (DSSAR) SNAP amplitude ratios were calculated in 22 controls and 46 patients with type 2 diabetes mellitus. Combined sensory scores (superficial peroneal, sural, dorsal sural, and medial plantar SNAPs), and amplitude ratio scores (SRAR, DSSAR, and SMFAR) were assessed. The parameters were compared statistically between the patient and control groups.

**Results:**

All SNAP amplitudes were significantly lower in patients as compared with those of the controls. Reduced medial plantar SNAP amplitude was the most frequent abnormality in the patient group. DSSAR and SMFAR, but not SRAR were found to have significant value in differentiating patients from controls with low sensitivity and moderate specificity. The combined sensory score improved the diagnostic accuracy for diabetic polyneuropathy, while the other combined scores add no additional value in this respect.

**Conclusion:**

Distal nerve conduction studies (NCSs) are most useful in diagnosing mild diabetic polyneuropathy. Although DSSAR and SMFAR can be moderately sensitive alternatives, particularly when used in combined scores, these ratios do not add any diagnostic value in patients with axonal polyneuropathies of similar severity.

## 1. Introduction

Length-dependent sensory-motor axonal polyneuropathy is the most common form of polyneuropathies in patients with diabetes mellitus (DM), eventually affecting nearly two-thirds of patients through a dynamic process, which becomes most prominent as the duration of illness prolongs [1]. Electrodiagnostic (EDX) methods play a crucial role in the diagnosis of length-dependent sensory-motor axonal polyneuropathy in DM [1,2]. The earliest and predominant finding in the EDX studies in these types of axonal polyneuropathies is sensory nerve conduction abnormalities. However, the amplitude of the sural sensory nerve action potential (SNAP), which is the most commonly used sensory nerve conduction study (NCS) in length-dependent axonal polyneuropathy, could stay within normal limits in some patients who have symptoms suggesting the presence of polyneuropathy and challenge the diagnosis [3–5]. For this reason, NCSs of sensory nerves located more distally, such as the dorsal sural and medial plantar nerves, and the amplitude ratios (AR) of SNAPs recorded over nerves with longer and shorter axons have been presented to increase the sensitivity of EDX studies, depending on the concept that nerves having longer axons are more severely affected [3–5]. The most frequently used method for the latter is the sural-to-radial amplitude ratio (SRAR) [6–9].

In 1995, Lee et al. introduced a technique for the evaluation of the medial femoral cutaneous sensory nerve in 32 healthy subjects using inguinal stimulation and recording 14 cm distal to the point of stimulation [10]. Then, in 2020, Castro et al. recorded SNAPs from 104 healthy subjects using a bar recording electrode 10 cm proximal to the medial edge of the patella, and stimulation was applied 14 cm proximal to the recording point [11]. All of these previous reports emphasized that medial femoral cutaneous SNAPs could easily be recorded in cases belonging to different age groups [10–13].

We evaluated the sural-to-medial femoral cutaneous amplitude ratio (SMFAR) which we obtained by comparing the SNAP amplitude of the medial femoral cutaneous nerve to the SNAP amplitude of the sural nerve far distally located on the same extremity, to use as a potentially more sensitive alternative to the SRAR in the diagnosis of length-dependent sensory-motor axonal polyneuropathy. Additionally, the dorsal sural-to-sural amplitude ratio (DSSAR) was calculated, which represents relatively distal and proximal segments of the same nerve. We aimed to compare the diagnostic sensitivities and specificities of SRAR, DSSAR and SMFAR with frequently used conventional sensory NCSs as well as with unconventional and more rarely used distal sensory NCSs in length-dependent sensory-motor axonal polyneuropathy in patients with DM.

## 2. Materials and methods

### 2.1 Participants

The present study was conducted in the EDX Laboratory in the Clinical Neurophysiology Department of Istanbul University from February 2020 to January 2022.

This study included 46 patients with type 2 DM followed-up in Diabetes and Neurology Outpatient Clinics at Istanbul University and who had symptoms and signs indicating the presence of polyneuropathy based on the Turkish version of the Michigan Neuropathy Screening Instrument (MNSI) (questionnaire score ≥3 and clinic score ≥2) [14,15].

Twenty-two controls from the staff of our departments and their relatives, who had not any symptoms and signs indicating a neurological disease were recruited prospectively after they agreed to participate in the study.

Exclusion criteria were previous hip and spinal surgery, surgery of the inguinal or the antero-medial thigh region, trauma to the inguinal region, traumatic nerve injuries of the studied nerves, lumbosacral radicular symptoms, neuromuscular disease, and systemic conditions known to be associated with polyneuropathy except those related to type 2 DM. The body mass index was not considered to be an exclusion criterion.

The study protocol conformed to the Helsinki Declaration of Human Rights and was approved by the local Ethics Committee (İstanbul University, 7/01/2020, number: 32). Written informed consent was obtained from all subjects who participated in the study.

### 2.2 Clinical evaluation

All patients underwent a detailed neurologic evaluation. Muscle strengths were scored according to the Medical Research Council sum score (MRCSS) [16]. To detect the clinical severity of polyneuropathy, all patients were administered the ‘Neuropathy Impairment Score in the Lower Limbs’ (NIS-LL) [17].

### 2.3 Electrodiagnostic (EDX) tests

EDX tests were performed unilaterally from the right side in patient and control groups using a Medelec Synergy device version 20.1.0 (Natus Medical, Inc., Middleton, Wisconsin, USA). The right side was chosen because of the layout of the study room. The temperature of the studied extremity was measured at the beginning and the end of each EDX study and held constant at ≥32 °C using hot water bags if necessary. Ulnar sensory NCS was studied using ring stimulation electrodes placed over the fifth finger and recording orthodromically with a bar electrode (inter-electrode distance = 3 cm) placed at the wrist 11 cm away from the stimulating electrode. Sural SNAP was recorded antidromically using a bar electrode placed posterior to the lateral malleolus and stimulated at the dorsal aspect of the midcalf 14 cm away from the recording electrode. Peak-to-peak amplitudes of the SNAPs were measured, and conduction velocities (CV) were calculated. Ulnar, tibial, and peroneal motor NCSs were performed using the belly-tendon method. Stimulation points and recording muscles were as follows: ulnar nerve, wrist, below and above the elbow, abductor digiti minimi muscle; tibial nerve, ankle and popliteal fossa, abductor hallucis muscle; peroneal nerve, ankle and fibular head, and extensor digitorum brevis muscle. At the most distal stimulation points, the distance between the active recording electrode and the cathode of the stimulating electrode was kept at 6.5 cm for the ulnar nerve and 8 cm for the tibial and peroneal nerves. The evaluated motor NCS parameters were compound muscle action potential (CMAP) base-to-peak amplitudes, distal motor latencies (DMLs), and CVs. Twenty F-waves were recorded with stimulation of the ulnar, peroneal, and tibial nerves over the wrist or ankle level, and minimum F-wave latencies were measured.

In EDX examinations, the abnormality of at least one of the other parameters (ulnar SNAP and CMAP amplitudes, tibial and peroneal CMAP amplitudes, ulnar, tibial, and peroneal DMLs, CVs or F-wave minimum latencies) together with the presence of low sural SNAP amplitude was considered as evidence of EDX polyneuropathy, defined by Dyck et al [18]. The limits for abnormality in NCS were defined according to the normal values of our laboratory.

#### 2.3.1. Radial sensory NSC and SRAR

The recording bar electrode was placed over the superficial radial nerve located over the tendon of the extensor pollicis longus traveling through the thumb. The nerve was stimulated over the ridge of the radius bone 10 cm away from the recording electrode [13]. SRAR was calculated by dividing the sural SNAP amplitude by the radial SNAP amplitude.

#### 2.3.2. Superficial peroneal, medial plantar, dorsal sural sensory NCSs and DSSAR

For superficial peroneal sensory NCS, the recording bar electrodes were placed over the lateral one-third of the line between the medial and lateral malleoli at the ankle. The superficial peroneal nerve was stimulated over the lateral calf, 12 cm proximal to the recording active electrode [13].

**Figure 1:**
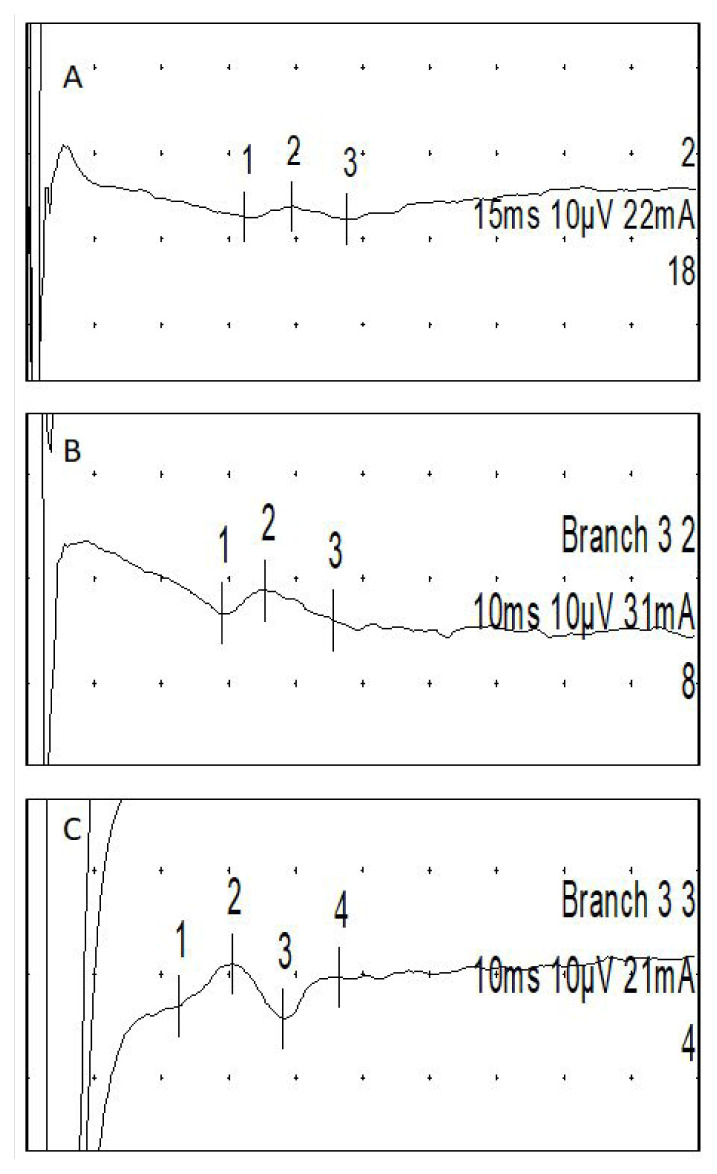
The examples of nerve conduction studies in a healthy subject. (A) Medial plantar sensory nerve action potenatial,15 ms/D, 10 μV/D. (B) Dorsal sural sensory nerve action potential, 10 ms/D, 10 μV/D. (C) Medial femoral cutaneous sensory nerve action potential, 10 ms/D, 10 μV/D.

Medial plantar SNAPs were recorded orthodromically by stimulating the big toe using a pair of ring electrodes and placing the recording active over the flexor retinaculum behind the medial malleolus (Figure 1 (A)).

The dorsal sural nerve was stimulated posterior to the lateral malleolus, 10 cm away from the recording active electrode placed over the lateral aspect of the foot on the line of the fifth finger (Figure 1 (B)) [13]. DSSAR was calculated by dividing the amplitude of dorsal sural SNAP to sural SNAP.

The limits of abnormality for radial and superficial peroneal sensory NCSs were defined according to the normal values of our laboratory. The studies for medial plantar and dorsal sural nerves were considered abnormal only when no response was recorded because of their typically low amplitude responses, which are difficult to elicit in some normal subjects and their liability to earlier amplitude loss in neuropathies [2,19].

#### 2.3.3. MFC NCSs and SMFAR

SNAPs were recorded using a bar recording electrode (active 10 cm proximal to the patella) along an imaginary line drawn between the medial edge of the patella and the femoral pulse below the inguinal ligament. Stimulation was applied 14 cm proximal to the recording point (Figure 1 (C)). A flat disc ground electrode was placed between the stimulation and recording sites [11]. SMFAR was calculated by dividing the sural SNAP amplitude by the MFC SNAP amplitude.

The lower limits of ARs (SRAR, DSSAR, and SMFAR) were determined using the 5^th^ percentile value of the healthy controls. The 5^th^ percentile indicates that 95% of the controls have a value above this limit. For each of the three ARs, the ratios were calculated with two different methods: 1) By giving the value zero for the unrecordable SNAPs in response to more distal nerve stimulation, and 2) by excluding the ARs of the unrecordable distal SNAPS from the statistical comparisons.

#### 2.3.4. Combined scores

With the aim of increasing diagnostic sensitivity of EDX studies, combined scores were calculated for both amplitudes and ARs as follows:


$$\begin{array}{l} \;\;\;\text{Combined sensory score}=\text{Superficial peroneal SNAP}\\ \text{amplitude}+\text{Sural SNAP amplitude}+\text{Dorsal sural SNAP}\\ \text{amplitude}+\text{Medial plantar SNAP amplitude}\\ \;\;\;\text{Combined AR score}=\text{SRAR}+\text{DSSAR}+\text{SMFAR}\\ \;\;\;\text{Combined AR score without SARA}=\text{DSSAR}+\text{SMFAR} \end{array}$$

### 2.5. Statistical analyses

Statistical analyses were performed using IBM Statistical Package for the Social Sciences (SPSS) SPSS Statistics for Windows, version 21.0 (IBM Corp., Armonk, N.Y., USA). Comparisons between the controls and the DM groups were assessed using the parametric or nonparametric tests according to the distribution of the data.

Categorical variables (abnormal sensory NCS) were compared using the Chi-square test between patients with DM with and without abnormal sural SNAP/abnormal EDX study. A receiver operating characteristic (ROC) curve was also constructed to evaluate the discriminative power of SRAR, DSSAR, and SMFAR between the patient and control groups. The effect of Body Mass Index (BMI) on NCSs were assessed using nonparametric correlation analysis. Statistical significance level was defined at p < 0.05.

## 3. Results

Demographic and clinical characteristics of the study population are presented in Table 1. The patient and the control groups did not differ with regard to age, whereas the mean BMI and weight were higher in patients with DM than those of controls. In the controls, there was no significant correlation between BMI and any of the EDX parameters. However, in the DM group, MFC amplitude exhibited a weak significant negative correlation with BMI (correlation coefficient −0.374, p = 0.011), while SMFAR did not.

The lower limits of SRAR, DSSAR, and SMFAR calculated using the 5^th^ percentile value of the healthy controls were 0.3, 0.06, and 1.5, respectively. In the control group, all sensory NCSs could be recorded, while MFC SNAPs were absent in seven patients with DM, in whom sural SNAPs could be recorded in four. The BMI of those four were 44, 43, 28, and 28.

The amplitudes of the ulnar, superficial peroneal, sural, dorsal sural, MFC, superficial radial, and medial plantar SNAP were found to be lower in patients with DM when compared to the controls (Table 2). Furthermore, the SRAR, DSSAR, and SMFAR, including those without zero values except DSSAR, were also found to be lower in the DM group than those of the controls (Table 2). In a similar vein, the combined sensory, AR, and total score was found to be lower in patients than in controls.

The percentage of abnormal SNAPs in patients with DM grouped according to the presence of abnormal sural SNAP are presented in Table 3. Medial plantar SNAP amplitude was the most frequently abnormal NCS (55%) in patients with normal sural SNAP, whereas patients with abnormal sural SNAP showed numerous abnormal NCS results (69%–92%).

The percentage of abnormal SRAR, DSSAR, and SMFAR in patient group was 30.4 (14/46), 42.1 (16/38), and 28.2 (11/36), respectively. SMFAR and DSSAR could not be calculated in 7 and 8 patients, respectively because of unrecordable SNAPS in response to more proximal stimulation.

Abnormal DSSAR and SMFAR were present in three and two patients with normal sural SNAP (11% and 15%, respectively). When the zero values were not included, one and two patients still had abnormal DSSAR and SMFAR, respectively. Abnormal SRAR, DSSAR, and SMFAR results were observed in 54%, 42% and 72% of the patients with abnormal sural SNAP, respectively (Table 3). Table 4 shows the abnormal sensory NCSs which were determined using our laboratory values in patient groups with normal and abnormal EDX study. Abnormal SRAR, DSSAR, and SMFAR were present in 2 (8%), 3 (14%), and 6 (24%) of the patients with a normal EDX study, respectively. The percentage of abnormal sensory NCSs and ARs, were higher in patients with abnormal sural NCS and those with an abnormal EDX study than in the patients with normal EDX study (Table 3 and 4).

**Figure 2:**
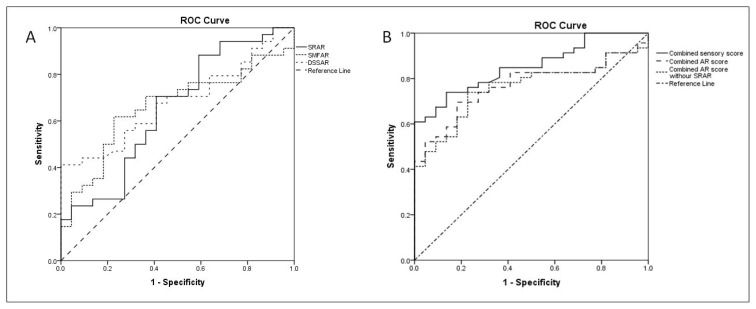
ROC curve analyses for SRAR, DSSAR, and SMFAR (A), and the combined sensory and AR scores (B) in discriminating control and patients with DM. AR, amplitude ratio; DM, diabetes mellitus; ROC, receiver operating characteristics; DSSAR, dorsal sural-to-sural amplitude ratio; SMFAR, sural-to-medial femoral cutaneous amplitude ratio; SNAP, sensory nerve action potential; SRAR, sural-to-radial amplitude ratio. The combined sensory score includes the superficial peroneal, sural, dorsal sural, and medial plantar SNAP amplitude. The combined AR score is the sum of SRAR, SMFAR, and DSSAR. The combined AR score without SRAR is the sum of SMFAR and DSSAR.

DSSAR and SMFAR, but not SRAR were able to distinguish patients from controls with low sensitivity and moderate specificity (Table 5 and Figure 2 (A,B)). The ROC curves for combined sensory and AR scores had higher area under curve (AUC) with high specificity and moderate sensitivity levels than single ARs (p≤0.001).

## 4. Discussion

In this study, we investigated various sensory NCSs including three ARs, SRAR, DSSAR and SMFAR, in the electrodiagnosis of length-dependent sensory-motor axonal polyneuropathy in DM. The main finding of the study was that, although the absolute ARs demonstrated significant differences between patients and controls, these ratios did not improve the diagnostic accuracy for distinguishing the patients from controls, compared with the combined sensory NCSs of the distal nerves in the lower extremity which already showed good sensitivity and specificity.

In many studies, it has been stated that medial dorsal cutaneous, dorsal sural, and medial plantar sensory NCSs increase the sensitivity of neuropathy diagnosis even in patients with normal EDX studies or in patients with impaired glucose intolerance [3–5,20–31]. Similarly, in our study, dorsal sural SNAP and medial plantar SNAP amplitudes were found to be abnormal even in patients with normal EDX study. The major concern related to the performance of these NCSs was the difficulty of recording in the healthy elder population, which was largely disproved in most studies [23,32,33].

Diabetic sensory polyneuropathy is a length-dependent axonal neuropathy affecting the lower extremity distal nerves at the earliest stages of the disease. There is good reason to assume that the ratio between a distal lower extremity SNAP amplitude to a proximal upper or lower extremity SNAP amplitude can give information even before routine NCSs become abnormal in detecting neuropathy. Previously, several studies investigated the most well-known ratio, SRAR [6,8,34,35] and one study for medial plantar mixed NAP-to-radial SNAP amplitude ratio (MPRAR) [9].

Ruthkove et al. reported that SRAR of <0.4 had a diagnostic sensitivity and specificity of 90% in examining mild axonal polyneuropathy, despite the presence of normal sural SNAP amplitude [6]. Esper et al. and Overbeek et al. suggested a limit of 0.21 for SRAR as a better cut-off value [34,36]. Rajabally et al. showed that SRAR of <0.21 had also a sensitivity of 52.1% and a specificity of 83.9% in patients with clinical large fiber sensory neuropathy and normal sural SNAP amplitude [37]. Sullivan et al. reported that the sensitivity of an SRAR value of 0.21 in detecting sensorial neuropathy in patients with normal sural SNAP amplitude was low [7]. Similarly, Barnett et al. reported that, when the cut-off value was set to 0.21, none of their patients with diabetic sensory polyneuropathy and with normal sural SNAP amplitudes had abnormal SRAR, providing no benefit to sural NCS [35]. Therefore, the diagnostic utility of SRAR remains obscure in the diagnosis of polyneuropathy in DM. MPRAR has been investigated in one study previously and found to be no superior to medial plantar mixed NAP alone although MPRAR is more sensitive than SRAR [9].

We hypothesized that using a proximal lower extremity SNAP relative to sural SNAP would increase the diagnostic sensitivity of the study. We used MFC NCS for this purpose because MFC is easy to record by surface electrodes and not prone to entrapment. Another AR was calculated using the dorsal sural and sural SNAP amplitudes to represent the distal and proximal segment of the same nerve. We observed that SRAR failed to distinguish patients from controls while SMFAR and DSSAR could differentiate the patients with DM from the controls with low sensitivity and moderate specificity. The combination of the three AR scores demonstrated an improvement in diagnostic accuracy, although this still remained inferior to the combined score of five distal sensory NCSs.

In order to ensure the reliability, we excluded the zero values for dorsal sural and sural NCSs in the calculation of the ARs. This is because these ARs are required in instances where the findings are equivocal, rather than when there is an obvious pathology. The high number of absent dorsal sural SNAPs in the patient group limits the utility of DSSAR. Abnormal rates for all ARs were also noticeably reduced in patient groups, particularly those with abnormal sural NCSs and EDX studies. This finding together with those mentioned above in the ROC analyses indicate that rather than the use of them in ARs, the absolute values of sensory NCSs provide superior results in diagnostic accuracy when multiple sensory nerves, particularly those located distally, are employed.

As expected, the rate of abnormal distal sensory NCSs were higher in patient groups with abnormal sural SNAP and with abnormal EDX study, which requires an abnormal sural and one another abnormal NCS, as described by Dyck et al [18]. Even though, a few patients had abnormal DSSAR and SMFAR while having normal sural NCSs and/or EDX study. On the other side, considering patients with DM with normal sural NCS, none had abnormal SRAR values. Although the sensitivities are low in the small number of patients presented herein, the newly proposed ARs can be an alternative to SRAR in selected patients with equivocal findings in DM.

MFC showed a weak negative correlation (correlation coefficient less than 0.4 [38]) with BMI in the patient group, similar to the findings of a previous study on healthy controls [11]. In addition, in four patients with DM with high BMI, MFC SNAP was unrecordable while sural SNAP still could be recorded. This seems to be a limitation in our patient group. However, considering that SMFAR was not correlated with BMI, it can still be used in EDX studies.

## 5. Conclusion

The findings of our study showed that the addition of lower extremity distal sensory nerve examinations to routine NCSs was the most useful method for the diagnosis of diabetic polyneuropathy, even in the period when there was no obvious EDX signs of neuropathy. ARs, particularly DSSAR, and SMFAR, may have a modest value in distinguishing patients with DM.

## Figures and Tables

**Table 1:** Demographic and clinic features of patients and controls.

	Controls (n = 22)	Patients with DM (n = 46)	p
Mean of age ± SD	51.3 ± 10.0	53.6 ± 7.7	0.401
Number of females/Number of males	13/9	24/22	0.615
BMI ± SD	27.0 ± 4.4	31.5 ± 4.4	**≤0.001**
Weight, kg ± SD	71.9 ± 12.2	85.8 ± 14.6	**≤0.001**
Duration of DM, year ± SD	-	10.9 ± 6.2	-
Duration of neuropathy symptoms, month ± SD	-	8.4 ± 6.4	-
HbA1c ± SD	-	8.5 ± 2.0	-
MRCSS ± SD	-	59.5 ± 1.3	-
NIS-LL ± SD	-	8.4 ± 6.4	-
MNSI questionnaire score ± SD	-	5.5 ± 2.0	-
MNSI clinic score ± SD	-	3.7 ± 1.6	-
MNSI total score ± SD	-	9.2 ± 2.8	-

BMI, body mass index; DM, diabetes mellitus; F, female; M, male; MRCSS, Medical Research Council Sum Score; NIS-LL, Neuropathy Impairment Score in the Lower Limbs; SD, standard deviation

**Table 2:** Electrodiagnostic data of sensory nerve conduction studies in patients and control.

	Controls (n = 22)	Patients with DM (n = 46)	p
Ulnar SNAP amplitude, μV ± SD	16.4 ± 5.8	9.6 ± 4.7	**≤0.001**
Sural SNAP amplitude, μV ± SD	13.9 ± 5.6	7.9 ± 5.7	**≤0.001**
Superficial peroneal SNAP amplitude, μV¤ ± SD	10.0 ± 5.3	4.6 ± 4.5	**≤0.001**
Dorsal sural SNAP amplitude, μV ± SD	5.8 ± 4.7	2.0 ± 2.5	**≤0.001**
Medial plantar SNAP amplitude, μV ± SD	1.9 ± 1.1	0.5 ± 1.1	**≤0.001**
Radial SNAP amplitude, μV ± SD	25.1 ± 9.8	18.6 ± 9.2	**0.010**
MFC SNAP amplitude, μV ± SD	3.9 ± 1.6	2.8 ± 2.3	**0.019**
SRAR¶ ± SD	0.6 ± 0.3	0.4 ± 0.3	**0.002**
SMFAR¶^ ± SD	4.0 ± 1.7	3.2 ± 3.7	**0.008**
DSSAR¶^ ± SD	0.4 ± 0.3	0.2 ± 2.2	**0.011**
SRAR¥ ± SD	0.6 ± 0.3	0.5 ± 0.2	**0.028**
SMFAR¥ ± SD	4.0 ± 1.7	3.7 ± 3.8	**0.042**
DSSAR¥ ± SD	0.4 ± 0.3	0.4 ± 0.2	0.973
Combined sensory score* ± SD	31.6 ± 12.2	15.0 ± 11.3	**≤0.001**
Combined AR score§ ± SD	5.0 ± 1.8	3.3 ± 3.7	**≤0.001**
Combined AR score without SRARϯ ± SD	4.4 ± 1.7	2.9 ± 3.6	**0.001**

AR, amplitude ratio; DM, diabetes mellitus; DSSAR, dorsal sural-to-sural amplitude ratio; SMFAR, sural-to-medial femoral cutaneous amplitude ratio; SNAP, sensory nerve action potential; SRAR, sural-to-radial amplitude ratio; SD, standard deviation

¤ Superficial peroneal sensory NCS data of one patient was missing.

¶ Amplitude ratios calculated by including the zero (unrecordable SNAPs in response to more distal nerve stimulation) values.

^ SMFAR and DSSAR could not be calculated in 7 and 8 patients, respectively because of unrecordable SNAPS in response to more proximal stimulation.

¥ Amplitude ratios calculated by excluding the zero values.

* Sum of superficial peroneal, sural, dorsal sural, and medial plantar SNAP

§ Sum of SRAR, SMFAR, and DSSAR

ϯ Sum of SMFAR and DSSAR

**Table 3:** The number of abnormal sensory nerve conduction studies in patients with a normal and abnormal sural SNAP.

	Normal sural SNAP amplitude (n = 20)	Abnormal sural SNAP amplitude (n = 26)	p
Abnormal superficial peroneal SNAP amplitude, n(%)¤	5 (26.3)	18 (69.2)	**0.007**
Abnormal dorsal sural SNAP amplitude, n(%)	2 (10.0)	21 (80.8)	**≤0.001**
Abnormal medial plantar SNAP amplitude, n(%)	11 (55.0)	24 (92.3)	**0.005**
Abnormal SRAR, n(%)	0 (0.0)	14 (53.8)	**≤0.001**
Abnormal SMFAR, n(%)	2 (11.1)	9 (42.9)	**0.037**
Abnormal DSSAR, n(%)	3 (15.0)	13 (72.2)	**0.001**
Abnormal SRAR¥, n(%)	0 (0.0)	6 (37.5)	**0.006**
Abnormal SMFAR¥, n(%)	2 (11.1)	4 (25.0)	0.387
Abnormal DSSAR¥, n(%)	1 (5.6)	0 (0.0)	1.000

DSSAR, dorsal sural-to-sural amplitude ratio; SMFAR, sural-to-medial femoral cutaneous amplitude ratio; SNAP, sensory nerve action potential; SRAR, sural-to-radial amplitude ratio

¤ Superficial peroneal sensory NCS data of one patient was missing.

¥ Amplitude ratios calculated by excluding the zero values

**Table 4:** The number of abnormal sensory nerve conduction studies in patients with abnormal and normal EDX study.

	Normal EDX study (n = 25)	Abnormal EDX study (n = 21)	p
Abnormal superficial peroneal SNAP amplitude, n(%)¤	9 (37.5)	14 (66.7)	0.075
Abnormal dorsal sural SNAP amplitude, n(%)	5 (20.0)	18 (85.7)	**≤0.001**
Abnormal medial plantar SNAP amplitude, n(%)	15 (60.0)	20 (95.2)	**0.006**
Abnormal SRAR, n(%)	2 (8.0)	12 (57.1)	**≤0.001**
Abnormal SMFAR, n(%)	3 (13.6)	8 (47.1)	**0.033**
Abnormal DSSAR, n(%)	6 (24.0)	10 (76.9)	**0.004**
Abnormal SRAR¥, n(%)	2 (8.7)	4 (36.4)	0.070
Abnormal SMFAR¥, n(%)	3 (13.6)	3 (25.0)	0.641
Abnormal DSSAR¥, n(%)	1 (5.0)	0 (0.0)	1.000

DSSAR, dorsal sural-to-sural amplitude ratio; SMFAR, sural-to-medial femoral cutaneous amplitude ratio; SNAP, sensory nerve action potential; SRAR, sural-to-radial amplitude ratio

¤ Superficial peroneal sensory NCS data of one patient was missing

¥ Amplitude ratios calculated by excluding the zero values.

**Table 5:** ROC curve analyses of the ARs and combined scores in discriminating DM patients and controls.

	Cut-off value	Sensitivity	Specificity	AUC	p	95 CI (upper-lower)
DSSAR	0.2	55.9	72.7	0.680	**0.024**	0.542–0.818
SMFAR	2.9	61.8	77.3	0.662	**0.042**	0.518–0.806
Combined sensory score	20.8	73.9	86.4	0.852	**≤0.001**	0.765–0.939
Combined AR score	3.7	69.6	81.8	0.768	**≤0.001**	0.657–0.879
Combined AR score without SRAR	3.3	73.9	77.3	0.759	**0.001**	0.646–0.872

AR, amplitude ratio; AUC, area under curve; CI, confidence interval; DM, diabetes mellitus; DSSAR, dorsal sural-to-sural amplitude ratio; ROC, receiver operating characteristic; SMFAR, sural-to-medial femoral cutaneous amplitude ratio; SRAR, sural-to-radial amplitude ratio.

## Data Availability

The data that support the findings of this study are available from the corresponding author upon reasonable request.
